# Determination of Asymmetric and Symmetric Dimethylarginine in Serum from Patients with Chronic Kidney Disease: UPLC-MS/MS *versus* ELISA

**DOI:** 10.3390/toxins8050149

**Published:** 2016-05-13

**Authors:** Jente Boelaert, Eva Schepers, Griet Glorieux, Sunny Eloot, Raymond Vanholder, Frédéric Lynen

**Affiliations:** 1 Department of Organic Chemistry, Separation Science Group, Ghent University, Krijgslaan 281, S4-bis, B-9000 Ghent, Belgium; jente.boelaert@gmail.com; 2 Department of Internal Medicine, Nephrology Section, Ghent University Hospital, De Pintelaan 185, B-9000 Ghent, Belgium; eva.schepers@ugent.be (E.S.); griet.glorieux@ugent.be (G.G.); sunny.eloot@ugent.be (S.E.); raymond.vanholder@ugent.be (R.V.)

**Keywords:** asymmetric dimethylarginine, symmetric dimethylarginine, UPLC-MS/MS, ELISA, chronic kidney disease

## Abstract

Asymmetric dimethylarginine (ADMA), an endogenous inhibitor of nitric oxide (NO) synthesis, and its structural isomer symmetric dimethylarginine (SDMA) are uremic toxins accumulating in chronic kidney disease (CKD) patients. The objective of this study was to develop and validate a robust UPLC-MS/MS method for the simultaneous determination of ADMA and SDMA in human serum. Chromatographic separation after butyl ester derivatization was achieved on an Acquity UPLC BEH C18 column, followed by tandem mass spectrometric detection. After validation, the applicability of the method was evaluated by the analysis of serum samples from 10 healthy controls and 77 CKD patients on hemodialysis (CKD5HD). Both ADMA (0.84 ± 0.19 µM *vs.* 0.52 ± 0.07 µM) and SDMA concentrations (2.06 ± 0.82 µM *vs.* 0.59 ± 0.13 µM) were significantly (*p* < 0.001) elevated in CKD5HD patients compared to healthy controls. In general, low degrees of protein binding were found for both ADMA and SDMA. In addition, an established commercially available ELISA kit was utilized on the same samples (*n* = 87) to compare values obtained both with ELISA and UPLC-MS/MS. Regression analysis between these two methods was significant (*p* < 0.0001) but moderate for both ADMA (*R* = 0.78) and SDMA (*R* = 0.72).

## 1. Introduction

Chronic kidney disease (CKD) is a worldwide public health problem with cardiovascular disease as the most important and often fatal complication [1,2]. A myriad of toxic solutes, normally cleared by the kidneys, among which asymmetric dimethylarginine (ADMA) and symmetric dimethylarginine (SDMA), accumulate in the body of CKD patients [3]. Both originate from proteolysis of methylated proteins [4]. Arginine residues within proteins can be post-translationally methylated by a class of enzymes, named protein arginine methyltransferases. Proteolysis of proteins containing methylated arginine releases free methylarginines into the cytosol. Once released, ADMA acts as an endogenous inhibitor of nitric oxide synthase by competing with L-arginine as the substrate [5]. Elevated plasma ADMA levels have been associated with endothelial dysfunction [5,6], which is an essential contributing element to vascular disease, and were found in patients with various risk factors for atherosclerosis such as in CKD [7,8]. Plasma ADMA levels may predict the progression of renal injury in patients with early-stage CKD [9,10], and are an independent risk factor for cardiovascular disease and all-cause mortality in different populations, such as patients with coronary artery disease [11,12] and patients with end stage renal disease [13,14]. SDMA, a structural isomer of ADMA, has long been considered biologically inactive [4,5]. Biologic activity was however at first suggested by the finding of a dose-responsive inhibition of NO synthesis by a mechanism different from that elicited by ADMA [15]. Subsequently, SDMA was shown to play a prominent role in leukocyte activation by enhancing generation of radical oxygen species, which is attributable to increased calcium influx via store-operated Ca^2+^ channels [16] and to activation of nuclear factor-κB resulting in cytokine production [17]. In addition, SDMA was proposed as biomarker for renal function outperforming creatinine-based equations for determining estimated glomerular filtration rate [18,19].

It has been suggested that the removal of ADMA in standard hemodialysis is completely hampered, eliciting the hypothesis that the compounds are protein bound [20]. Other studies confirmed that removal of dimethylarginines is lower than would be expected with regard to their molecular weight [21,22]. However, in the latter studies the decrease in ADMA was obviously more substantial than in the one by Kielstein *et al.*, [20]. Hence, investigation of the protein-binding of ADMA and SDMA is essential to shed light on these inconsistent results.

Since ADMA shows a very narrow range of normal concentrations, even a small increase in its concentration might be linked to cardiovascular risk. Therefore, high analytical precision is of extreme importance to discriminate between normal and slightly elevated concentrations. Current methods for determination of ADMA and SDMA in biofluids include gas chromatography coupled with mass spectrometry (GC-MS) [23,24], high performance liquid chromatography (HPLC) with fluorescence detection (HPLC-FLD) after derivatization [25,26,27,28,29,30,31,32], HPLC with mass spectrometric detection (LC-MS and LC-MS/MS) underivatized [13,33,34,35,36,37,38,39,40,41] or after derivatization [42,43,44,45,46], and capillary electrophoresis coupled with ultraviolet (CE-UV) [47] or with mass-spectrometric detection (CE-MS) [48]. In addition, an enzyme-linked immunosorbent assay (ELISA) has been developed [49] and several comparisons between this assay and chromatographic methods have been described for ADMA [49,50,51,52,53,54,55]. Some comparisons suggest that the enzyme-linked immunosorbent assay (ELISA) assay for ADMA suffers from matrix effects producing concentration-dependent positive bias compared with other methods [50,51,52]. Moreover, some discrepancies seem to exist between the reported method comparisons. For SDMA no method comparisons have been described yet.

In this study, the primary aim was to develop and validate a robust ultra (high) performance liquid chromatography (U(H)PLC)-MS/MS method for the simultaneous determination of ADMA and SDMA in human serum. Secondly, we investigated the protein binding of ADMA and SDMA in serum. Finally, the UPLC-MS/MS data were compared with an established ELISA for both ADMA and SDMA. The UPLC-MS/MS method with the MRM detection was chosen because of its power to measure specific compounds in a very accurate way with a minimum of interferences and its possibility to perform high throughput analysis. The latter method was compared to ELISA because this method can be easily introduced in a research setting.

## 2. Results

**Figure 1:**
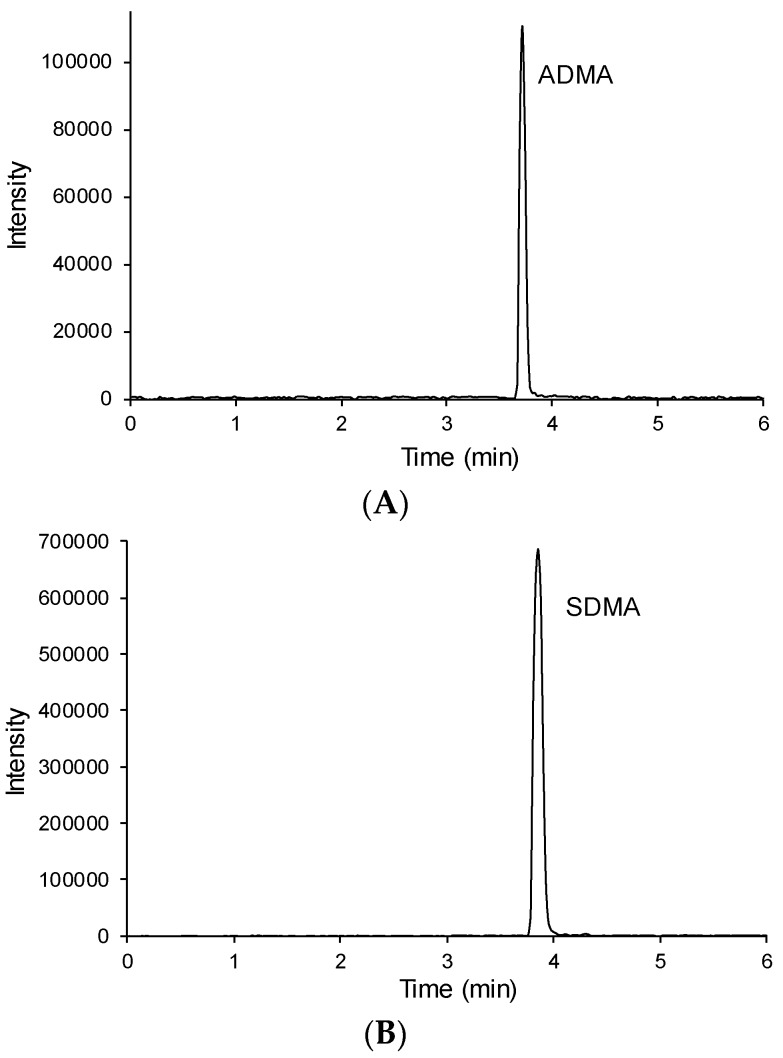

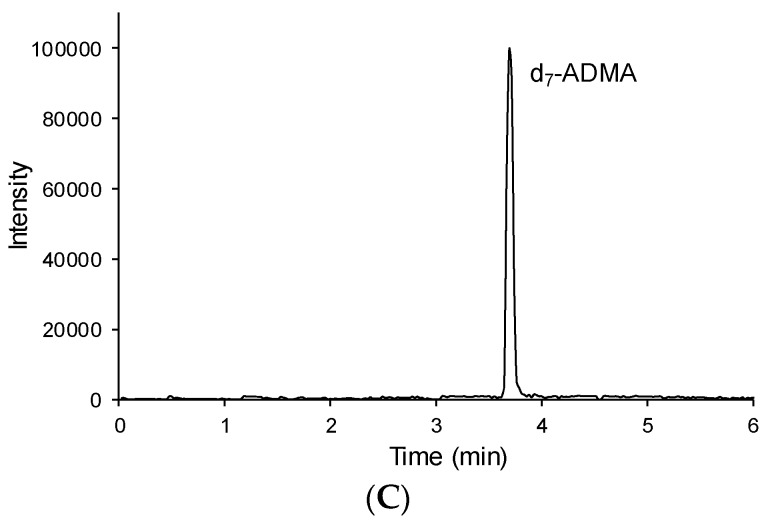
Representative chromatograms obtained simultaneously via the UPLC-MS/MS method from a CKD patient on hemodialysis for ADMA (mass transition 259/214) (**A**) and SDMA (mass transition 259/228) (**B**) and for the internal standard (mass transition 266/221) (**C**).

To promote retention on the Acquity UPLC BEH C18 column and to improve sensitivity, the dimethylarginines were derivatized to their butyl ester analogues. This derivatization was based on the method described by Schwedhelm *et al.*, [46], strengthening the selectivity of the method. Different mobile phase compositions were compared to achieve retention, separation, symmetric peak shapes, method robustness and fast analysis. Ammonium acetate buffer solution (5 mM, pH 4.3) was mixed with 15% methanol (containing 0.1% acetic acid) as initial mobile phase. Detection was performed by tandem mass spectrometry operated in multiple reaction monitoring (MRM) mode, which is characterized by its sensitivity and selectivity and therefore widely implemented in bioanalysis. ADMA and SDMA exhibit the same protonated molecular ion with mass-to-charge ratio (*m*/*z*) of 259 and have their most intense mass transition in common (259 → 70). Next to this transition they have unique mass transitions, which have lower intensity. ADMA fragments by loss of 45 (corresponding to dimethylamine) and SDMA by loss of 31 (corresponding to methylamine). Although ADMA and SDMA were almost baseline separated and it was therefore not absolutely necessary to distinguish them by their different fragmentation pattern, we chose to monitor these unique transitions because of the unequivocal selectivity towards the parent compounds. The sensitivity for the dimethylarginines was further optimized by adjustment of the cone and collision energy potentials (Table 1). Representative MRM chromatograms obtained from a CKD5HD patient are depicted in Figure 1. No interferences from other endogenous substances were apparent.

After optimization, the figures of merit of the method were established. The results of the accuracy, within- and between-day precision, recovery, limit of detection (LOD) and limit of quantification (LOQ) tests are summarized in Table 2. The LOD and the LOQ were below the lowest calibration point of the seven-point calibration curves (*i.e.*, below 0.1 µM). Good linearity (*r*^2^ ≥ 0.99) was observed for both dimethylarginines using a least square fit. Isotopically labeled ADMA served as internal standard for both ADMA and SDMA. The deviation of the mean measured concentration of the quality control (QC) samples from the theoretical concentration was below 12.35%. Within- and between-day precision were below 3.48% and 10.93%, respectively. Recoveries were high and more importantly reproducible. The effect of the matrix on signal intensity was below 15% for all 6 serum samples. The occurrence of a significant matrix effect could therefore be excluded. Moreover, the use of the isotopically labeled internal standard, the gradient to 100% mobile phase A (0.1% acetic acid in methanol), and the additional isopropanol wash after every 15 injections were all measures reducing the risk for such effect.

Patient and control characteristics are displayed in Table 3. Hemodialysis patients were routinely dialyzed for 245 ± 18 min, with a blood flow of 321 ± 37 mL/min (QB range 220–350 mL/min) and QD = 500 mL/min in hemodialysis mode with high flux dialyzer, or in postdilution hemodiafiltration mode.

**Figure 2:**
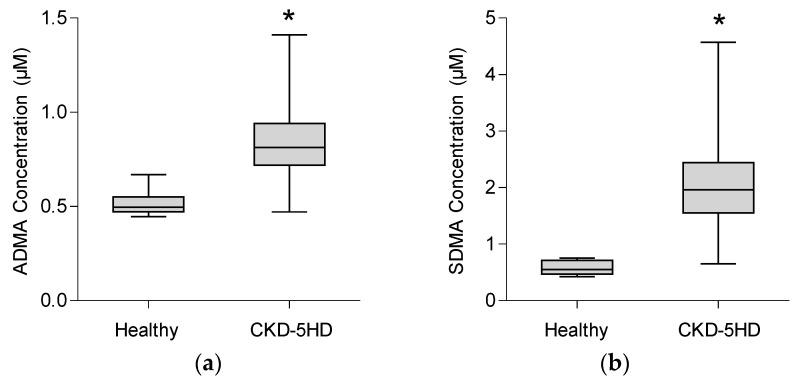
ADMA (**a**) and SDMA (**b**) serum concentrations determined by UPLC-MS/MS in healthy controls (*n* = 10) and CKD5HD patients (*n* = 77), * *p* < 0.001 *versus* healthy.

Figure 2 displays the dimethylarginine serum concentrations determined by UPLC-MS/MS in healthy controls and CKD5HD patients. ADMA and SDMA concentrations are both significantly elevated in CKD patients compared to healthy controls (*p* < 0.001). For ADMA, we found a mean concentration of 0.52 ± 0.07 µM in healthy controls and 0.84 ± 0.19 µM in CKD5HD patients. For SDMA, mean normal concentration is 0.59 ± 0.13 µM and mean concentration in CKD5HD patients is 2.06 ± 0.82 µM. The control values are within the previously reported ranges for ADMA [50] and slightly higher for SDMA [27,35]. Our reported concentrations for CKD patients are also consistent with published values [27,56].

In general, low degrees of protein binding were found for both ADMA and SDMA. Protein binding of ADMA is 6.53% ± 4.93% in healthy controls and 4.01% ± 2.90% in hemodialysis patients. For SDMA protein binding is 12.02% ± 8.73% in healthy controls and 10.36% ± 9.74% in hemodialysis patients. It was, however, not possible to determine the phenomenon accurately as most of the concentration differences were smaller than the error margins of the method. An unpaired *t*-test showed that the total concentrations were significantly higher (*p* < 0.0001) than the free concentrations in all conditions. A significant increase in ADMA and SDMA was also seen in hemodialysis patients *versus* healthy controls for both total and free concentrations. For SDMA, however, quite high interindividual variability in protein binding was encountered. Basic compounds such as dimethylarginines might preferentially bind to alpha1-acid glycoprotein (AAG) [57], an acute phase protein that is often elevated in CKD [58]. In order to obtain more information on the interindividual differences, the correlation between protein binding and AAG concentration was investigated. However, no correlation between these variables was found. Nevertheless, we can conclude that ADMA and SDMA are only minimally protein bound in contrast to what is described by Kielstein *et al.*, [20] and that therefore, it is more likely that the removal of ADMA in standard dialysis is hampered because of complex kinetics and distribution rather than of protein binding as recently suggested by Schepers *et al.*, and Sitar *et al.*, [59,60].

Next to the developed UPLC-MS/MS method, a commercially available ELISA assay was performed in parallel to determine ADMA and SDMA in the same 87 serum samples. This assay provided mean normal concentrations of 0.49 ± 0.06 µM for ADMA and 0.62 ± 0.09 µM for SDMA. Mean concentrations of 0.97 ± 0.23 µM for ADMA and 2.09 ± 0.59 µM for SDMA were found in CKD5HD patients. In a previous study by our group [17], we already found in a cross-sectional analysis of 142 patients in consecutive stages of CKD, using the same ELISA assays, that both ADMA and SDMA increase with decrease in renal function.

**Figure 3:**
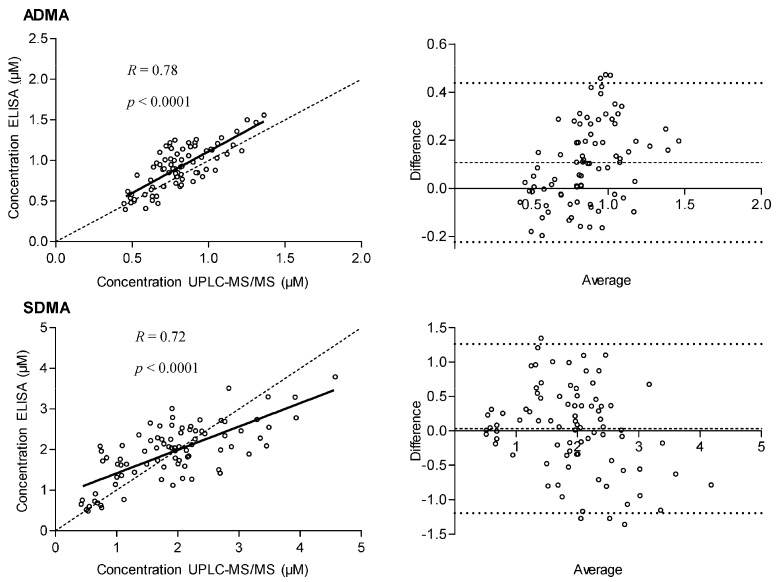
Correlation between concentrations determined by ELISA and UPLC-MS/MS (**left**). The dashed line is the identity line. Bland-Altman plots for ADMA and SDMA: comparison of ELISA and UPLC-MS/MS assay (**right**).

UPLC-MS/MS and ELISA results showed only moderate correlation, with *R* = 0.78 for ADMA and *R* = 0.72 for SDMA. In literature, different method comparisons have been described for ADMA determination only. Schulze *et al.*, reported for the first time on the ELISA assay for ADMA [49]. To assess the analytical performance of the assay, ELISA was compared with a GC-MS and LC-MS/MS method [49]. Good correlations were found for both GC-MS (*R* = 0.991, *p* < 0.0001) and LC-MS/MS (*R* = 0.984, *p* < 0.0001). However, in three out of the nine samples submitted to the GC-MS method, serum was spiked with ADMA concentrations exceeding the concentrations found in human serum of CKD patients. In spite of the good correlation, an overestimation of ~20% was observed for the serum ADMA concentrations determined by ELISA compared with LC-MS/MS. Subsequently, several independent groups also compared their methods with the commercially available ELISA for ADMA. Valtonen *et al.*, found no correlation between serum ADMA concentrations determined by HPLC-FLD (orthophtaldialdehyde (OPA)-derivatization) and the ELISA assay [55]. However, Schulze *et al.*, signaled that the ELISA kit controls were outside the given range in two out of three ELISA kits [53]. Martens-Lobenhoffer *et al.*, found considerable disagreement in the Bland-Altman plot between LC-MS (OPA-derivatization) and ELISA for ADMA concentrations in plasma from healthy and diseased individuals [51]. The ELISA assay appeared to overestimate the ADMA concentrations by a factor of about two. The matrix dependence of the ELISA was suggested as cause of this overestimation. Široká *et al.*, found good correlation (*R* = 0.944, *p* < 0.0001) for plasma ADMA concentrations between HPLC-FLD (OPA-derivatization) and the ELISA assay [54]. However, the ELISA assay provided about two-fold higher ADMA concentrations than HPLC-FLD. Horowitz and Heresztyn found a linear relationship with an *R*^2^ = 0.69 between ADMA concentrations (serum and plasma) determined by HPLC-FLD (AccQ-Fluor™ derivatization, Waters, Milford, MA, USA) and ELISA [50]. However, the difference between the two methods increased with increasing ADMA concentration, as was also the case in the study by Martens-Lobenhoffer *et al.*, [54]. Pecchini *et al.*, compared plasma ADMA concentrations determined by LC-MS and ELISA (*R* = 0.69) and again an increasingly pronounced overestimation in ADMA levels by ELISA was found with increasing ADMA concentration [52]. After checking normality and linearity of the residuals, linear regression analysis of the concentrations measured in the present study was performed. As shown in the left panel of Figure 3 we could demonstrate that, although moderate, a significant linear relationship (*p* < 0.0001) exists between ELISA and the UPLC-MS/MS method for both ADMA (*R* = 0.78) and SDMA (*R* = 0.72). Based on Bland Altman graphs the two methods tend to show dissimilarity with increasing concentrations. In general, ELISA tends to overestimate ADMA concentrations (Figure 3, right panel). Moreover, the difference between the two methods tends to increase with increasing ADMA concentration, which is in agreement with previous findings. For SDMA no method comparisons have been described yet and no reference frame is therefore available. From the Bland-Altman plot a slightly inversed trend seems visible for SDMA, but no fixed bias could be shown.

By comparison, the new proposed UPLC-MS/MS method, which allows simultaneous determination of both arginine derivatives in the CKD range while ensuring robustness due to the chromatographic separation, is shown to be efficient and applicable in CKD research. The observed and reported discrepancies related to the ELISA assay warrant further study.

## 3. Conclusions

In conclusion, a robust UPLC-MS/MS has been developed and validated for the simultaneous determination of ADMA and SDMA in serum. The method has been applied to analyze serum from healthy controls and CKD patients on hemodialysis. A significant increase in serum concentrations was found in hemodialysis patients. Protein binding of both ADMA and SDMA has been investigated and low protein binding was suggested, therefore in the future only total concentration of both compounds needs to be considered. Accuracy and precision testing confirm the effectiveness of the UPLC-MS/MS methodology. Comparison between the developed UPLC-MS/MS method and the commercially available ELISA showed a moderate correlation, this needs to be taken into account when considering absolute concentrations. When estimating changes in concentrations e.g., during dialysis therapy, both methods are applicable.

## 4. Materials and Methods

### 4.1. Chemicals

Asymmetric dimethylarginine (ADMA) and symmetric dimethylarginine (SDMA) were obtained from Merck (Darmstadt, Germany). The internal standard 2,3,3,4,4,5,5-d_7_-ADMA:HCl:H_2_O (d_7_-ADMA, 98%) was purchased from Cambridge Isotope Laboratories, Inc. (Andover, MA, USA). Methanol and water, both LC-MS grade, were purchased from Biosolve (Valkenswaard, The Netherlands). Acetic acid was obtained from Panreac (Barcelona, Spain). Ammonium acetate was purchased from Fluka (Bornem, Belgium). 1-Butanol was obtained from Merck (Darmstadt, Germany) and hydrochloric acid from Fluka (Bornem, Belgium). ELISA kits were purchased from DLD Diagnostika GmbH (Hamburg, Germany).

### 4.2. UPLC-MS/MS Assay

#### 4.2.1. Instrumentation

The Waters UPLC-MS/MS system comprised an Acquity UPLC System and a Quattro Micro triple quadrupole mass spectrometer (Milford, MA, USA). Separation was performed on an Acquity UPLC BEH C18 column (1.7 µm, 2.1 mm × 100 mm) with an Acquity UPLC BEH C18 VanGuard precolumn (1.7 µm, 2.1 mm × 5 mm). The mobile phase consisted of 0.1% acetic acid in methanol (mobile phase A) and 0.1% acetic acid in 5 mM ammonium acetate (mobile phase B, pH 4.3). A gradient elution at a flow of 0.25 mL/min was performed with an initial composition of 15% A, which was held for 3.5 min, followed by an increase in 0.01 min to 100% A (for 1.5 min) and finally a re-equilibration (5 min). The total run time was 10 min. The column was flushed with isopropanol every 15 runs to remove phospholipids which can be a significant source of imprecision in quantitative analyses [61]. The column temperature and the autosampler temperature were kept at 21 °C and 8 °C, respectively. Mass spectral ionization, fragmentation, and acquisition parameters were optimized on the tandem quadrupole mass spectrometer using electrospray ionization (ESI) in the positive mode (Table 1). The ion source temperature and the desolvation temperature were maintained at 120 and 350 °C. Nitrogen was used as nebulizer and desolvation gas. The desolvation gas flow was set at 650 L/h and the cone gas flow was 10 L/h. Capillary voltage was 3260 V. The collision gas (argon, purity 99.999%) was set at 3.44 × 10^−1^ Torr. Quantification was performed in the multiple reaction monitoring (MRM) mode with dwell and interscan delay times of 0.2 and 0.1 s, respectively. Data were acquired and processed using Masslynx software (version 4.0, Waters, Milford, MA, USA).

#### 4.2.2. Preparation of Stock Solutions, Calibration Standards and Quality Control (QC) Samples

Stock solutions of ADMA (1.24 mM), SDMA (1.24 mM) and d_7_-ADMA (4.78 mM) were prepared in water for HPLC (Merck). An internal standard solution containing 2.39 µM d_7_-ADMA was obtained from the stock solution by dilution with H_2_O. This solution was further used for spiking all calibration solutes and the samples such that they contained a final concentration of 0.60 µM d_7_-ADMA, at injection. Calibration curve standards were prepared at 0.1, 0.25, 0.49, 0.74, 0.99, 1.24 and 1.48 µM for ADMA and at 0.1, 0.25, 0.49, 1.24, 2.47, 3.71 and 4.94 µM for SDMA. Low, medium, and high-concentration quality-control (QC) samples were prepared by combining three samples of normal serum, selected because of their low levels of endogenous uremic toxins, and spiking them with appropriate amounts of dimethylarginines, taking into account the endogenous baseline level. The low, medium, and high QC samples were spiked with, respectively, 0.25, 0.49 and 0.99 µM ADMA and 0.25, 1.24 and 3.71 μM SDMA. All calibration standards and QC samples were freshly prepared on the day of analysis and were run in triplicate. All stock solutions were stored at −20 °C and were stable at these conditions.

#### 4.2.3. Sample Preparation

The sample preparation procedure described by Meert *et al.*, was slightly adapted [62]. Blood samples from healthy controls and CKD5HD patients allowed to clot and were subsequently centrifuged. Serum was collected, frozen and stored at −80 °C. Serum samples were thawed at room temperature and vortex mixed to ensure homogeneity. Subsequently, 40 µL of internal standard solution (2.39 µM of d_7_-ADMA) was added to 160 µL of serum, vortex mixed and 600 µL of water was added. To determine the total concentration, serum samples were first deproteinized by heat denaturation. To this end, samples were heated for 30 min at 90 °C. After heating, the samples were placed on ice for 10 min. All serum samples were then ultrafiltered using Millipore Centrifree ultrafiltration devices (MWCO 30,000 Da, Millipore, Billerica, MA, USA) at 1469× *g* for 25 min. To determine the free fraction, serum samples were filtered through Millipore Centrifree ultrafiltration devices prior to heating. Subsequently, 600 µL of ultrafiltrate was dried under nitrogen at room temperature. ADMA, SDMA and the internal standard were analyzed as their butyl ester derivatives. Derivatization step was performed by dissolving the dried extract in 500 µL of a freshly prepared 1 M HCl in 1-butanol solution. After 2 min vortexing, the solution was kept at 70 °C for 20 min. The solvent was subsequently removed by evaporation under nitrogen. The derivatized samples were reconstituted in 120 µL of the initial mobile phase and were transferred to an autosampler vial (glass insert P/N WAT094171). Afterwards, 20 µL was injected on the column. Samples were prepared on the day of analysis.

#### 4.2.4. Validation

The tested validation parameters were selectivity, LOD, LOQ, linearity, accuracy, precision, recovery, and matrix effects. The limits of detection were determined according to the EPA recommended procedure [63]. A standard solution containing each dimethylarginine at the estimated signal to noise (S/N) of 10 was injected seven times, and the standard deviations of the peak areas (and of the corresponding concentrations) were calculated. The limits of detection were calculated by multiplying the standard deviations by three. The limit of quantification was calculated as three times the LOD. Seven point calibration curves were generated with aforementioned concentrations (Section 4.2.2). Quantification was carried out by internal standard calibration with d_7_-ADMA as internal standard for both ADMA and SDMA. The accuracy and precision of the method were evaluated by the analysis of spiked samples at three QC levels (*n* = 5). Accuracy was defined as the difference between the calculated and the specified amount for the selected compound and expressed as a percentage. Precision was obtained as the percentage relative standard deviation (% RSD) for a selected compound and level. The relative recovery (%) was determined by comparing the peak area ratios of ADMA and SDMA in normal serum samples spiked before heat denaturation to the peak area ratios of normal serum spiked after ultrafiltration, respectively. To evaluate matrix effects, we spiked water and six different serum samples at the low QC level and compared the areas obtained in water with those of the spiked serum samples.

#### 4.2.5. Application to Biological Samples

The described method was applied to serum samples from healthy controls (*n* = 10) and from CKD patients on hemodialysis (CKD5HD, *n* = 77). In order to establish protein binding, both total (T) and free (F) concentrations were determined for the healthy control group and for 20 randomly selected CKD5HD patients. The degree of protein binding was calculated as follows: (T − F)/T × 100. The present study was approved on 10 June 2010 by the local Ethics Committee (Belgian registration number: (B67020107926), Ghent University Hospital, Ghent, Belgium), and performed in accordance to the Declaration of Helsinki. Written informed consent was obtained from all participants.

### 4.3. ELISA Assay

Two established competitive ELISA’s were used for measuring ADMA and SDMA according to manufacturer’s guidelines. Briefly, serum samples were acylated before adding to the microtiter plate. Overnight, acylated ADMA or SDMA competes with the solid phase bound ADMA or SDMA for a fixed number of rabbit anti-ADMA or anti-SDMA antiserum binding sites. After equilibration, antibody bound to the solid phase ADMA or SDMA is detected by the reaction of anti-rabbit peroxidase and the substrate TMB (3,3′,5,5′-Tetramethylbenzidine). Samples were analyzed using the EL808 Ultra Microplate Reader from Bio-Tek Instruments (Winooski, VT, USA) at 450 nm (reference wavelength of 650 nm) using the KC4V3.0 Analysis Software (Bio-Tek® Instruments, INC., Winooski, VT, USA). The amount of antibody measured is inversely proportional to the ADMA or SDMA concentration.

### 4.4. Nephelometric Assay

Alpha1-acid glycoprotein (AAG), an acute phase protein with a MW of 43 kDa, was determined by nephelometry on a Siemens Dade Behring Nephelometer (Siemens Healthcare Diagnostics Products GmbH, Marburg, Germany).

### 4.5. Statistical Analysis

Normality was checked and results are expressed as means ± standard deviations. Statistics were performed using GraphPad Prism 6.0 (GraphPad Software, Inc., La Jolla, CA, USA) via correlation analysis, unpaired *t*-test and Bland-Altman plots. A *p*-value of <0.05 was considered as statistically significant.

## Figures and Tables

**Table 1:** Detection settings for the investigated compounds.

Compound	Structure	*t*_R_ (min)	Transition (after Derivatization)	Cone (V)	Collision Energy (eV)
ADMA	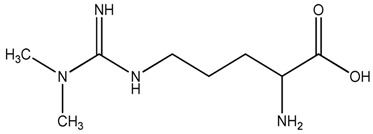	3.7	259 > 214	27	15
SDMA	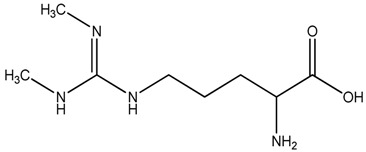	3.9	259 > 228	27	15
2,3,3,4,4,5,5-d_7_-ADMA	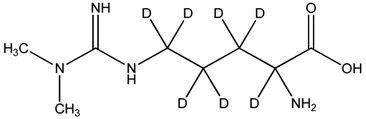	3.7	266 > 221	27	15

ADMA: asymmetric dimethylarginine; SDMA: symmetric dimethylarginine; 2,3,3,4,4,5,5-d_7_-ADMA isotopically labeled asymmetric dimethylarginine (internal standard); *t*_R_: retention time.

**Table 2:** Figures of merit.

Uremic Toxin	LOD (nM)	LOQ (nM)	QC Concentration Added (µM)	Accuracy (%)	Within-Day Precision (%)	Between-Day Precision (%)	Recovery (%)
ADMA	7.9	23.7	0.25	−9.65	2.00	7.79	100.5 ± 3.3
			0.49	−8.14	3.48	6.13	102.2 ± 3.2
			0.99	−4.87	2.56	5.25	98.7 ± 2.5
SDMA	6.4	19.2	0.25	−12.35	3.34	9.92	96.2 ± 4.8
			1.24	−6.83	2.31	10.93	94.6 ± 5.5
			3.71	11.69	1.93	8.61	97.4 ± 4.1

LOD: limit of detection; LOQ: limit of quantification; QC: quality control samples.

**Table 3:** Characteristics of controls and patients.

Characteristics	Healthy Controls	CKD5HD Patients
Number	10	77
Female	6	32
Age	61.4 ± 11.2	69.6 ± 12.4
Body weight (kg)	69.2 ± 15.9	71.2 ± 16.3
Dialysis vintage (months)	n.a.	39.2 ± 23.1
Diabetes	0	31

CKD5HD: Chronic kidney disease stage 5 on hemodialyis; n.a.: not applicable.

## Abbreviations

The following abbreviations are used in this manuscript:

ADMA	asymmetric dimethylarginine
SDMA	symmetric dimethylarginine
CKD	chronic kidney disease
CKD5HD	CKD patients on dialysis
HPLC	high performance liquid chromatography
UPLC	Ultra performance liquid chromatography
ELISA	enzyme-linked immunosorbent assay
GC	gas chromatography
MS	mass spectrometry
FLD	fluorence detection
CE	capillary electrophoresis
UV	ultraviolet
*m*/*z*	mass-to-charge ratio
LOD	limit of detection
LOQ	limit of quantification
QC	quality control
AAG	a1-acid glycoprotein
OPA	orthophtaldialdehyde
S/N	signal to noise
RSD	relative standard deviation
